# RNA-binding protein CELF6 modulates transcription and splicing levels of genes associated with tumorigenesis in lung cancer A549 cells

**DOI:** 10.7717/peerj.13800

**Published:** 2022-07-26

**Authors:** HuSai Ma, GuoWei Liu, Bin Yu, Joshua Wang, YaLi Qi, YiYing Kou, Ying Hu, ShunJun Wang, Fei Wang, Dong Chen

**Affiliations:** 1 Department of thoracic Surgery, Qinghai Provincial People’s Hospital, Xining, Qinghai Province, China; 2 Department of Thoracic Surgery, Qinghai Red Cross Hospital, Xining, Qinghai Province, P. R. China; 3 Wuhan Ruixing Biotechnology Co. Ltd., Wuhan, Hubei Province, China; 4 Department of Respiratory Medicine, Qinghai Provincial People’s Hospital, Xining, Qinghai Province, China; 5 Qinghai University School of Medicine, Qinghai University School of Medicine, Xining, Qinghai Province, China; 6 Qinghai University, Xining, Qinghai Province, China

**Keywords:** RNA-Binding Protein, CELF6, Alternative splicing, Lung cancer, RNA-seq

## Abstract

CELF6 (CUGBP Elav-Like Family Member 6), a canonical RNA binding protein (RBP), plays important roles in post-transcriptional regulation of pre-mRNAs. However, the underlying mechanism of lower expressed CELF6 in lung cancer tissues is still unclear. In this study, we increased CELF6 manually in lung cancer cell line (A549) and utilized transcriptome sequencing (RNA-seq) technology to screen out differentially expressed genes (DEGs) and alternative splicing events (ASEs) after CELF6 over-expression (CELF6-OE). We found that CELF6-OE induced 417 up-regulated and 1,351 down-regulated DEGs. Functional analysis of down-regulated DEGs showed that they were highly enriched in immune/inflammation response- related pathways and cell adhesion molecules (CAMs). We also found that CELF6 inhibited the expression of many immune-related genes, including TNFSF10, CCL5, JUNB, BIRC3, MLKL, PIK3R2, CCL20, STAT1, MYD88, and CFS1, which mainly promote tumorigenesis in lung cancer. The dysregulated DEGs were also validated by reverse transcriptase quantitative polymerase chain reaction (RT-qPCR) experiment. In addition, CELF6 regulates the splicing pattern of large number of genes that are enriched in p53 signaling pathway and apoptosis, including TP53 and CD44. In summary, we made an extensive analysis of the transcriptome profile of gene expression and alternative splicing by CELF6-OE, providing a global understanding of the target genes and underlying regulation mechanisms mediated by CELF6 in the pathogenesis and development of lung cancer.

## Introduction

Lung cancer is a malignant tumor disease, which has the second highest morbidity (11.4%) and the highest mortality (18%) rates among cancers worldwide according to global cancer statistics 2020 (Sung et al., 2021). It is estimated that from 2019 to 2020, there were more than 2,200,000 newly diagnosed cases and about 1,800,000 deaths in the world (Sung et al., 2021). Non-small cell lung cancer (NSCLC) is the most common type of lung cancer, and lung adenocarcinoma (LUAD) is the most common subtype of NSCLC, accounting for 40% of lung cancer cases (Duma, Santana-Davila & Molina, 2019). Although great progress has been made in the diagnosis and treatment methods in the past decades, the survival rate of patients is still very low (Miller et al., 2019). Currently, several traditional methods are being adopted to diagnose lung cancer, which is difficult to be detected in the early stage (Mottaghitalab et al., 2019). This may be the most important reason for the high mortality for lung cancer patients. Therefore, the determination and application of molecular markers and therapeutic targets are very important in improving the effectiveness of NSCLC treatment.

In recent years, scientists have begun to pay attention to the functions of RNA-binding proteins (RBPs) in diseases. RBPs refer to a large family of proteins that can bind to double-stranded or single-stranded RNAs and form ribonucleoprotein complexes in cells, playing key roles in transcriptional and post-transcriptional regulation. RBPs extensively affect the overall life cycle of RNAs, including production, modification, splicing, stabilization, transportation, intracellular localization, translation, and degradation (Perez-Perri et al., 2021; Xiao et al., 2019). Through this mechanism, RBPs play a very important role in the development of various diseases (Siang et al., 2020). To date, about 1,500 RBPs have been reported in the human genome (Gerstberger, Hafner & Tuschl, 2014). Recent studies have shown that 223 RBPs selected from the cancer genome atlas (TCGA) database may be involved in tumorigenesis, progression, invasion, and metastasis of LUAD (Li et al., 2020a; Li et al., 2020b; Li et al., 2020c). For example, RBM10 inhibits LUAD progression mainly by modulating the alternative splicing of EIF4H exon 5 (Zhang et al., 2020). RBM47 inhibits non-small cell lung carcinoma metastasis by regulating the stability of AXIN1 mRNA (Shen et al., 2020). KHSRP promotes the cell growth in tumors and metastasis of lung cancer (Yan et al., 2019). However, the functions of large number of RBPs are currently not clear.

CUGBP Elav-Like Family Member 6 (CELF6), located on chromosome 15, is a canonical RBP belonging to the CELF/BRUNOL protein family. Three RNA recognition motif (RRM) domains are found in CELF6, including two N-terminal and one C-terminal domains. CELF6 is strongly expressed in the kidney, brain, and testis, and is expressed at very low levels in most other tissue (Ladd et al., 2004). Functional researches revealed that CELF6 can regulate the alternative splicing of mRNA precursors, and may also be involved in regulating the stability of mRNA (Rieger et al., 2020). CELF6 could regulate muscle-specific splicing and enhancer-dependent alternative splicing (Ladd et al., 2004). A recent study reported that CELF6 may be a potential tumor suppressor, and may regulate cell proliferation and cell cycle progression by affecting the stability of p21 (Liu et al., 2019). In triple-negative breast cancer, CELF6 up-regulates the expression of FBP1 by stabilizing the mRNA expression of FBP1 and suppresses the development of breast cancer. Its over-expression inhibits proliferation, migration, and invasion of breast cancer cells (Yang et al., 2020). In our previous study, we found that the transcriptional level of CELF6 in A549 cells was significantly increased compared with in the control group after ultra-hypoxic treatment (Wang et al., 2021). Therefore, it is likely that CELF6, as an RBP, has important regulatory functions in lung cancer, which has not been previously investigated.

In the present study, we over-expressed CELF6 (CELF6-OE) in A549 cells derived from human NSCLC and set empty plasmid as negative control. After collecting cells, we performed high-throughput transcriptome sequencing (RNA-seq) to identify differentially expressed genes (DEGs) and alternative splicing genes (ASGs) after CELF6-OE, and explored their functions by analyzing the functional pathways and classifications of these genes. The present results demonstrated that CELF6 could regulate the expression and alternative splicing (AS) of genes associated with tumorigenesis, especially those involved in apoptosis and P53 signaling pathways, providing novel insight for the current understanding of CELF6 in regulating gene transcription and AS of NSCLC.

## Materials and Methods

### CELF6 expression in tumor samples

To have a global view of CELF6 expression pattern and its association with survival time in lung cancer, we used two web-based tools, including GEPIA2 (Tang et al., 2019) and KM-plotter (Gyorffy, 2021) to perform the analysis of gene expression and cancer survival using public datasets, including the cancer genome atlas (TCGA). The abbreviations of tumor types could be found at the website of GEPIA2.

### The culture of A549 cells and plasmid transfections

The identified human NSCLC cell line A549 cells with STR analysis were obtained from Procell (CL-0016, Wuhan, Hubei, China). Before cell culture, the A549 cells were detected to prevent mycoplasma contamination by the provider and our laboratory.

Then the A549 cells were cultured under traditional cell culture conditions which have been described detailedly in a previous study (Ma et al., 2020). CELF6 sequences were embedded in pIRES-hrGFP-1a plasmid (VT1056; YouBio, Shanghai, China). CELF6-OE and empty plasmids were transfected into A549 cells using Lipofectamine 2000 (Invitrogen, Carlsbad, CA, USA) following the manufacturer’s protocol. After 48 h cultivation, transfected cells were harvested for following experiments.

### Validation of CELF6 over-expression in A549 cells

We performed reverse transcription and quantitative polymerase chain reaction (RT-qPCR) and Western blot (WB) experiments to assess the expression level of CELF6 in OE and NC cells. Complementary DNA (cDNA) synthesis was done by standard procedures and RT-qPCR was performed on the Bio-Rad S1000 with Hieff™ qPCR SYBR® Green Master Mix (Low Rox Plus; YEASEN, Shanghai, China). Primer sequences for RT-qPCR were shown in Additional File 1. The relative level of each transcript was normalized to GAPDH mRNA level (internal control) using 2^−ΔΔCT^ method (Livak & Schmittgen, 2001). Raw and processed data of RT-qPCR results were shown in Additional File 2. Two-way ANOVA method was used to calculate statistical significance.

### Western blot to assess CELF6 protein level

A549 cells were treated by a published protocol (Ma et al., 2020). The treated samples were then boiled for 10 min in water with 1× SDS buffer and separated on 10% SDS-PAGE. Then samples were treated with TBST buffer (5% non-fat milk power and 20 mM Tris-buffered saline and 0.1% Tween-20) for 1 h at room temperature. Membranes were then incubated with primary antibody: FLAG antibody (1:1,000 dilution; polyclonal antibody; cat. no. 2368S; CST), GAPDH (1:2,000; ABclonal; Wuhan, Hubei, China) and then incubated with HRP-conjugated secondary antibody. Bound secondary antibody (anti-mouse or anti-rabbit 1:10,000) (Abcam) was detected using the enhanced chemiluminescence (ECL) reagent (Bio-Rad, Hercules, CA, USA, 170506). The uncropped gels for WB were presented in Fig. S1.

### RNA-seq preparation and data analysis

After cell culture, we extracted polyadenylated RNAs and constructed RNA-seq library according to published protocols (Song Ba et al., 2021). Briefly, we used TRIZOL (Ambion) method to extract total RNA and removed genomic DNA using RQ1 DNase (RNase free; Promega, Madison, WI, USA). After checking the quality and quantity of purified RNA, we used 10 μg total RNA to prepare a directional RNA-seq library by capturing polyadenylated mRNAs. Three biological replicates were prepared for both CELF6-OE and NC groups. The libraries were prepared according to the manufacturer’s instructions for high-throughput sequencing. Finally, we got 151-bp pair-end sequences for each sample. Low quality sequencing reads (containing N bases and quality score less than 20) and adaptor sequences from raw sequences were removed using FASTX-Toolkit (Version 0.0.13). Too short reads (<16 nt) were dropped. Next, quality-filtered reads were aligned to the GRCH38 genome by TopHat2 (Kim et al., 2013), with no more than four mismatches allowed. Reads with unique genomic locations were used for gene reads number counting and FPKM calculation (fragments per kilobase of transcript per million fragments mapped) (Trapnell et al., 2010). We calculated the replication and experimental power using R package RNASeqPower (Therneau, Hart & Kocher, 2022). The final power value 0.99 was used to detect a 2-fold expression. Then, the R Bioconductor package edgeR (Robinson, McCarthy & Smyth, 2010) was utilized to identify DEGs with the criteria *p*-value < 0.01 and fold change >1.5 or <2/3.

### CELF6-regulated alternative splicing events (RASEs) analysis

The AS events (ASEs) and CELF6-regulated AS events (RASEs) between CELF6-OE and control were analyzed using the ABLas pipeline (Xia et al., 2017b). Ten types of ASEs were analyzed, including alternative 5′ splice site (A5SS), alternative 3′ splice site (A3SS), cassette exon, exon skipping (ES), A5SS&ES, A3SS&ES, intron retention (IR), mutually exclusive exons (MXE), mutually exclusive 5′UTRs (5pMXE), and mutually exclusive 3′UTRs (3pMXE). To assess CELF6-regulated ASEs (RASEs), Student’s *t*-test was performed to calculate the statistical significance of the ratio difference between CELF6-OE and control samples. *p*-value < 0.05 and ratio difference >0.2 were considered criteria for RASEs.

### Functional enrichment analysis

To explore enriched functions of DEGs and RASGs, we used KOBAS 2.0 server (Xie et al., 2011) to identify enriched Gene Ontology (GO) terms and KEGG pathways. Hypergeometric test and Benjamini-Hochberg FDR were used to calculate the significant *p*-values and FDR values for each term or pathway.

### Reverse transcription qPCR validation of DEGs and RASEs

We also used RT-qPCR experiment to elucidate the DEG and RASG validity of the RNA-seq data. The details of DEG and RASG primers were presented in Additional File 1. For RASEs, we used a method to design specific PCR primers covering the junction boundary of constitutive exon and alternative exons (Liu et al., 2020). Total RNA of the cultured CELF6-OE and control A549 cells was used for validation. The relative expression levels of all the DEGs and RASGs were normalized to the internal control GAPDH. Raw and processed data of RT-qPCR results were shown in Additional File 2. Two-way ANOVA method was used to calculate statistical significance.

### Raw sequencing data

The raw sequencing data discussed in this manuscript are available under GEO series accession number GSE185865.

## Results

### CELF6 expression pattern in tumor samples from TCGA

**Figure 1:**
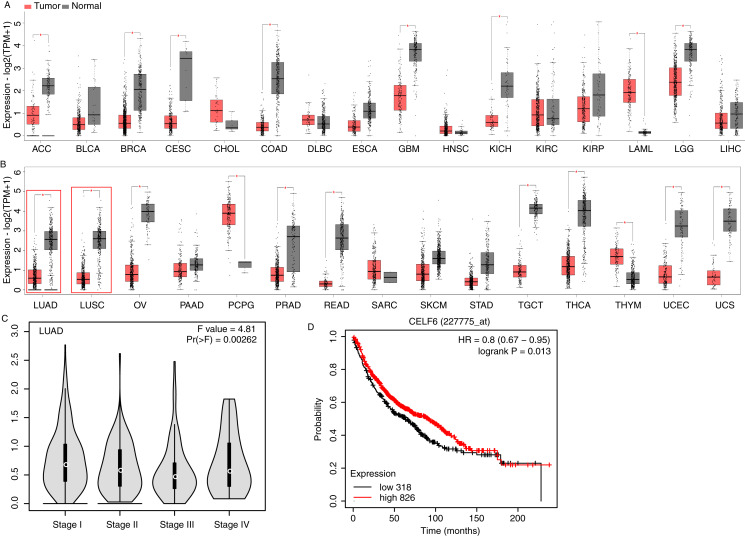
Expression pattern and prognosis analysis of CELF6 in multiple tumor and lung cancer samples. (A) Box plot of CELF6 expression levels (TPM) in 16 tumor types from TCGA database. *p-value < 0.05, unpaired Student’s *t*-test. The abbreviation of these tumor types could be found from the TCGA database (https://www.cancer.gov). (B) Box plot of CELF6 expression levels (TPM) in another 15 tumor types from TCGA database. *p-value < 0.05, unpaired Student’s *t*-test. Two lung cancer types were highlighted by red frame. (C) Violin plot showing the expression level change of CELF6 in the four disease stages of LUAD patient samples. Statistical difference was performed by one-way ANOVA method. (D) The Kaplan-Meier plot showing the prognosis difference between LUAD patients with high and low CELF6 expression levels.

To investigate CELF6 expression pattern and its influence on prognosis in lung cancer, we used GEPIA2 (Tang et al., 2019) and KM-plotter (Gyorffy, 2021) to analyze gene expression level and survival time using public datasets. CELF6 had significantly higher expression levels in normal tissue compared with in tumor tissue in 16 out of the 31 cancer types, whereas significantly higher expression levels of CELF6 in tumor tissue were only observed in LAML and PCPG (Figs. 1A and 1B). We found that the expression of CELF6 was significantly down-regulated (FDR < 0.05) in lung tumor samples (LUAD and LUSC) (Fig. 1B). We then detected its expression changes in disease stages of LUAD and LUSC patients. The result showed significant differences among the four stages (Fig. 1C), although the overall expression levels of CELF6 were very low (median TPM < 1). Furthermore, KM-plotter server was used to comprehensively explore the association between CELF6 expression and survival rate of LUAD patients. The results of log-rank test demonstrated that higher CELF6 level is associated with the longer overall survival (OS) time in LUAD patients (Fig.1B).

### CELF6 over-expression experiment was conducted in A549 cells

**Figure 2:**
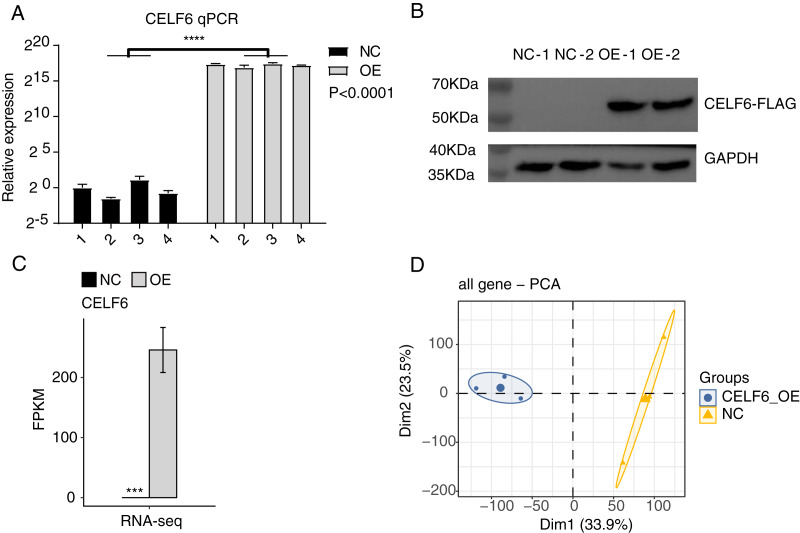
High quality sequencing data were obtained for expression and alternative splicing analysis. (A) The histogram showed the RT-qPCR results of control and CELF6-OE samples. Four biological replicates per sample. ****p < 0.0001, Student’s *t*-test. (B) The result of western blot experiment showed that the CELF6 overexpression was successful. (C) The expression level of CELF6 was up-regulated after overexpression based on RNA-seq (three biological replicates per sample). Error bars represent mean ± standard error of mean (SEM). *** *p* < 0.001, Student’s *t*-test. (D) Principal Component Analysis (PCA) based on FPKM values of all genes showed the sample correlation.

To explore the functions of CELF6 in lung cancer, a cell model was constructed by using cell culture and transfecting pIRES-hrGFP-1a-CELF6 into A549 cells to enhance CELF6 expression level. In order to assess the CELF6 over-expression (CELF6-OE) result in A549 cells, we detected CELF6 mRNA expression by RT‑qPCR and CELF6 protein expression by Western blot. The mRNA level of CELF6 was elevated significantly in transfected cells (*p* < 0.001, two-tailed *t*-test, Fig. 2A). The protein level of CELF6 was also elevated in transfected cells (Fig. 2B). These results suggest that CELF6-OE A549 cell models were successfully constructed. After collecting cells, cDNA libraries on CELF6-OE and control cells (three biological replicates per sample) were constructed for RNA‑seq experiment. The expression level of CELF6 was up-regulated after over-expression based on RNA-seq (*p* < 0.001, two-tailed *t*-test, Fig. 2C). Principal component analysis (PCA) based on expression values of all expressed genes showed that CELF6-OE was the major factor globally influencing the gene expression pattern (Fig. 2D).

### CELF6 over-expression resulted in some transcriptional differences

To investigate CELF6-mediated transcriptional regulation, we performed a comprehensive analysis of the RNA‑seq dataset. The sequencing result yielded about 83 million paired‑end raw reads per sample. After initial quality control steps, about 81 million high‑quality reads were generated per sample. We then found that about 97% of the reads from each sample could be aligned to the GRCh38 genome using TopHat2, and about 94% of the aligned reads from each sample had a uniquely aligned genomic location in the GRCh38 genome. We used uniquely mapped reads to calculate the normalized transcription level as FPKM, and detected 28,121 expressed genes from the six RNA‑seq datasets.

**Figure 3:**
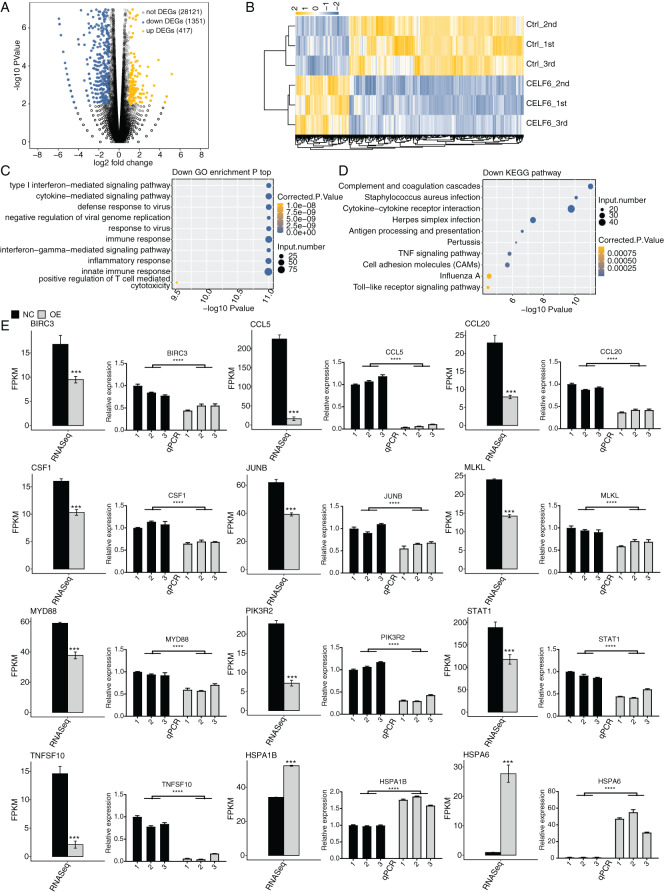
Differential expression analysis revealed that CELF6 downregulates large number of inflammation genes. (A) The number of differentially expressed genes (DEGs) based on the standard *p*-value < 0.01 and fold change ≥ 1.5 or ≤ 2/3. Up-regulated genes are labeled in red, whereas down- regulated are labeled in blue in the volcano plot. (B) The expression levels heatmap of all DEGs and reflected the change of genes expression. (C,D) Bar plot exhibited the most enriched KEGG pathways results of the Down-regulated and down-regulated DEGs. (E) Bar plot showing the expression pattern and statistical difference of DEGs. Reverse transcription qPCR validation of DEGs regulated by CELF6 in cancer cells; black bars are for the control group and grey bars for CELF6 overexpression. ***p-value < 0.001; ****p-value < 0.0001.

Based on the expression level of all detected genes from the CELF6-OE and control samples, we used strict criteria, absolute fold change ≥1.5 or ≤2/3 and FDR < 0.05 together with edgeR package, to identify differentially expressed genes (DEGs) that were potentially regulated by CELF6 at transcriptional level (Robinson, McCarthy & Smyth, 2010). In total, 417 up-regulated and 1,351 down-regulated DEGs were identified (Additional File 3). Volcano plot of all expressed genes displayed that CELF6-OE had a preference to repress gene expression in A549 cells (Fig. 3A). Hierarchical clustering heat map analysis of the DEG expression levels showed high regulatory consistency among the three replicates in both CELF6-OE and control samples (Fig. 3B). These results demonstrated that CELF6 has the potential to extensively regulate gene expression pattern in A549 cells.

### CELF6-OE preferentially down-regulated the expression of large number of inflammation genes

To further decipher the potential biological functions of these DEGs, we performed GO and KEGG enrichment analyses for both up- and down-regulated DEGs. The top 10 GO biological process (BP) terms enriched by up-regulated DEGs were shown with transport, axon guidance, signal transduction, cell adhesion, small molecule metabolic process, and cellular protein metabolic process. Up-regulated DEGs enriched in KEGG analysis were shown with MAPK signaling pathway, Estrogen signaling pathway, ECM-receptor interaction, protein processing in endoplasmic reticulum and so on. (Fig. S2, details in Additional Files 4 and 5). We then focused our attention on down-regulated DEGs. Strikingly, we found that the down-regulated DEGs were mostly associated with type I interferon-mediated signaling pathway, cytokine-mediated signaling pathway, immune response, interferon-gamma-mediated signaling pathway, inflammatory response, and innate immune response (Fig. 3C). According to the parallel KEGG analysis results (details in Additional File 6), down-regulated DEGs were significantly enriched in cytokine-cytokine receptor interaction, TNF signaling pathway, and cell adhesion molecules (CAMs) (Fig. 3D, details in Additional File 7), which were associated with immune response, cell migration and proliferation (Chen & Mellman, 2017; Makrilia et al., 2009). We then selected DEGs in A549 cells for further validation by RT‑qPCR. A total of twelve DEGs, including HSPA6, HSPA1B, TNFSF10, CCL5, JUNB, BIRC3, MLKL, PIK3R2, CCL20, STAT1, MYD88, and CFS1, were selected. The results showed high consistency with the RNA‑seq data (Fig. 3E). These results together suggest that CELF6 regulates DEGs associated with cancer immunity and progression.

### CELF6 modulates AS events of apoptosis-related genes in A549 cells

**Figure 4:**
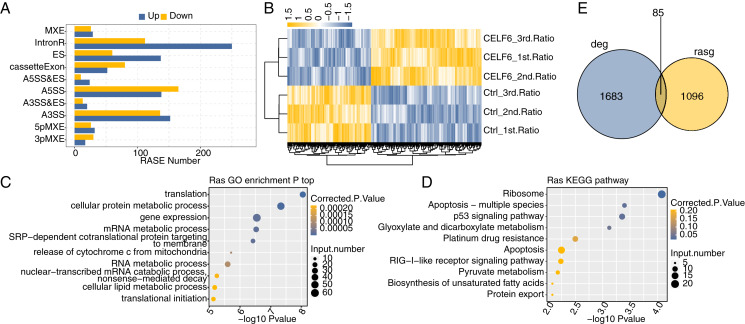
Identification and functional analysis of CELF6-regulated alternative splicing events in A549 cells. (A) The bar plot showing the number of all significant regulated alternative splicing events (RASEs). X-axis: RASE number. Y-axis: the different types of AS events. (B) Hierarchical clustering heat map of all significant RASEs based on splicing ratios. (C,D) Bar plot exhibited the most enriched GO biological process and KEGG pathways of the regulated alternative splicing genes (RASGs). (E) Venn diagram shows the result of overlap analysis between CELF6-regulated differentially expressed genes (DEGs) and alternative splicing genes (RASGs).

One definite aim of this study is to gain insight into the alternative splicing regulatory role of CELF6. Transcriptome data were used to examine regulated alternative splicing events (RASEs) by CELF6-OE in A549 cells. Among the uniquely aligned reads from CELF6-OE and control cells, about 38.94–40.41% were splicing junction reads. Of the 367,321 annotated exons from the human genome, 249,163 were detected. Using TopHat2 software (Kim et al., 2013), we detected 163,082 annotated and 162,218 novel splicing junctions, accounting for 49.8% of the total junctions (details in Additional File 8). Using the self-developed ABLas software (Xia et al., 2017a), we conducted overall analysis of and classified splicing methods on the results of splice junctions detected by TopHat2, and conducted statistics on various variable splicing events. A total of 17,691 known RASEs (annotated by reference genome) were identified, accounting for 26.3% of the total. The results showed that 49,565 novel splicing events (excluding intron retention) were identified, accounting for 73.7% of the total (details in Additional File 8). These results suggested that splicing profile is more complex than previously hypothesized. For each RASE, we calculated the differential splicing patterns between CELF6-OE and control samples. We found that 462 significant RASEs were identified (AS ratio difference ≥0.2 and *P*‑value ≤0.05). In detail, these RASEs included 47 3pMXE, 58 5pMXE, 288 A3SS, 33 A3SS&ES, 34 A5SS&ES, 303 A5SS,197 ES,55 MXE, 132 cassette exon and 362 intron retention events (Fig. 4A, details in Additional File 9). Hierarchical clustering heat map of all significant RASEs based on PSI showed high consistency with the CELF6-mediated post-transcription in both data sets (Fig. 4B). GO functional enrichment analysis demonstrated that the alternative spliced genes were enriched in translation, cellular protein metabolic process, gene expression, mRNA metabolic process, RNA metabolic process, cellular lipid metabolic process, and translational initiation (Fig. 4C). Enriched KEGG pathways (*p* < 0.05) included those involved in apoptosis-multiple species, p53 signaling pathway, platinum drug resistance, and apoptosis (Fig. 4D). The data suggested that CELF6 globally regulated ASEs in A549 cells. Excluding the changes in ASEs attributed to transcriptional regulation. Genes whose expression levels and AS were both regulated by CELF6 were also examined, and 85 genes were shared between the DEGs and RASGs (Fig. 4E). In addition, the differential splicing genes are enriched in p53 signaling pathway and apoptosis, which are closely related to tumorigenesis of lung cancer. From these differential genes, we screened out several genes associated with the occurrence of lung cancer. We chose CD47, CD44, FN1, CASP8, TP53, BAX, and SEC31A to identify CELF6-regulated alternative splicing events (Fig. S3). These results suggested that CELF6-OE markedly regulated AS of apoptotic-related genes.

### Validation of CELF6 RASEs associated with apoptosis in A549 Cells

**Figure 5:**
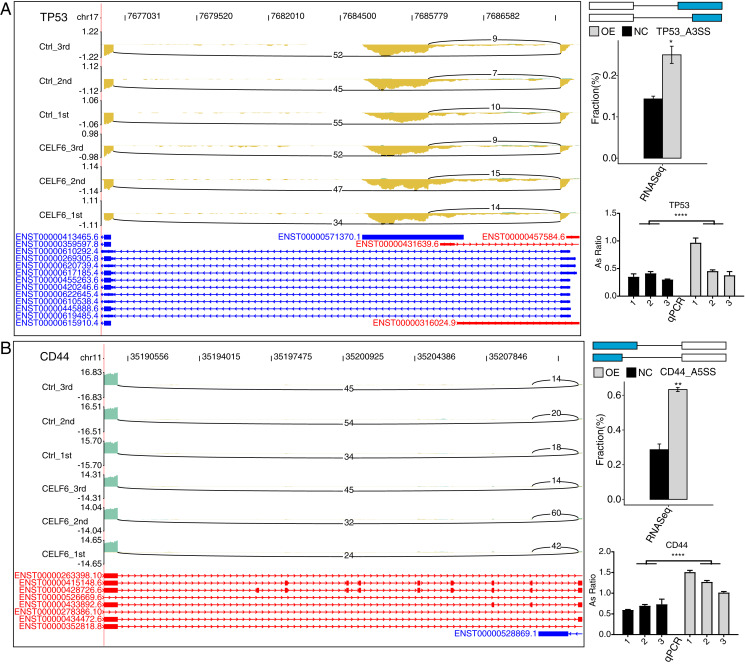
CELF6 regulated alternative splicing of TP53 and CD44. CELF6 regulated the alternative splicing event of TP53 (A) and CD44 (B) Left panel: IGV-sashimi plot showing the regulated alternative splicing events and binding sites across mRNA. Reads distribution of RASE is plotted in the up panel and the transcripts of each gene are shown below. Right panel: The schematic diagrams depict the structures of ASEs. RNA-seq validation of ASEs are shown at the bottom of the right panel. Error bars represent mean ± SEM. ****p-value < 0.0001; **p-value < 0.01; *p-value < 0.05; Student’s *t*-test.

To validate CELF6-regulated AS pattern of apoptotic genes, RT-qPCR experiments were conducted. The results showed that five AS events out of seven RASGs related to apoptosis and ECM receptor interaction showed high agreement with the transcriptome analysis results of RNA-seq data (Figs. 5A, 5B and S3). Two important ASEs were originated from TP53 and CD44 (Figs. 5A and 5B), which have been well studied as key genes in cell proliferation in cancers.

## Discussion

In this study, the role of CELF6 was studied to investigate how CELF6 regulates DEGs and ASEs in A549 cells. Using GEPIA2 software, 16 of the 33 cancer types from TCGA show the down-regulation of CELF6, indicating that CELF6 may play an important role in the pathogenesis and progression of various tumors. To study its potential mechanism in non-small cell lung cancer, we used the A549 cell line as a model to analyze the consequences of CELF6 over-expression. CELF6-OE was observed to negatively regulate the expression of many genes related to cancer inflammation. In addition, we observed that CELF6-OE significantly regulates the AS of genes involved in the P53 signaling pathway and the apoptosis pathway. These results revealed the role of CELF6 in the transcriptional and post-transcriptional regulation of lung cancer progression.

Previous research reports stated that CELF6 is generally down-regulated in colon cancer and breast cancer, and acts as a tumor suppressor (Liu et al., 2019; Yang et al., 2020). Over-expression of CELF6 inhibits tumor cell proliferation, colony formation, cell migration and invasion by directly binding to the 3′UTR of FBP1 and stabilizing FBP1 transcripts. CELF6 regulates cell proliferation and cell cycle progression *via* modulating p21 stability (Liu et al., 2019). Recent research demonstrated that the minor allele “C” of a single-nucleotide polymorphism in CELF6 is associated with increased risk of cervical cancer (Fang et al., 2017). CELF6 is associated with the repression of its CNS targets *via* the 3′ UTR *in vivo* (Rieger et al., 2020). Based on the identified functions of CELF6, we predict that it could also influence the phenotypes of lung cancer cells by acting as a tumor suppressor. Further experiments are needed to confirm this prediction. Meanwhile, other cell lines of lung cancer also need to be included in the following studies to further validate our conclusion.

In this study, GEPIA2 and KM-plotter software were used to analyze TCGA data and the results indicated that CELF6 showed lower expression levels in most tumor types compared with in matched normal tissue. Two lung cancer types (LUAD, lung adenocarcinoma; LUSC, lung squamous cell carcinoma) have lower CELF6 expression levels, too. In this study, it is speculated that CELF6 may function as a tumor suppressor in NSCLC.

The tumor necrosis factor (TNF)/TNF receptor pathway affects the survival of cancer patients, and the potentially functional genetic variations of TNF/TNFR signaling pathway genes affect the survival rate of NSCLC patients (Guo et al., 2019). The TNF-α/NF-κB pathway plays an important role in tumor cell invasion and metastasis (Wu & Zhou, 2010). Recent studies have shown that Sotetsuflavone can inhibit the epithelial-mesenchymal transition, invasion, and metastasis of lung cancer cells in A549 cells through the TNF-α/NF-κB pathway (Wang et al., 2018). Therefore, TNF pathway is a significant biological progression in non-small cell lung cancer. In this study, we found that CELF6 can negatively regulate the expression of large number of inflammation-related genes, which are mainly enriched in the TNF signaling pathway, including CCL5, JUNB, BIRC3, MLKL, PIK3R2, CCL20, TNFAIP3, and CFS1. CC Motif Chemokine Ligand five is a member of the super family of secreted proteins involved in immune regulation and inflammation in the formation of chemokines. It can increase lung cancer migration through PI3K, Akt and NF-κB pathways (Huang et al., 2009). Over-expression of JunB Proto-oncogene increases the involvement of EMT in the invasion and metastasis of lung cancer (Wanna-Udom et al., 2020); Colony-stimulating factor 1 (CSF1) increases the proliferation and invasion of lung cancer cells (Hung et al., 2014). CCL20 is a family of secreted proteins in immune regulation and inflammatory processes, and promotes the migration and proliferation of lung cancer cells (Wang et al., 2016). These genes are related to the occurrence and progression of NSCLC, including cell proliferation, invasion, and metastasis. Their downstream targets are regulated by CELF6, which implies that CELF6 may affect the progression of NSCLC by regulating the expression of these genes. Further research is needed to confirm and verify the association between CELF6 and TNF signaling pathways. For example, WB can be used to detect changes in the protein levels of the above-mentioned inflammatory genes at the cell level of CELF6-OE.

Here we note that the empirical genes of the ASEs regulated by CELF6 are mainly located in the genes associated with p53 signaling pathway, apoptosis, and ECM receptor interaction. Associated genes were TP53 (Tumor Protein P53), CD44 (Cell Surface Glycoprotein CD44), FN1 (Fibronectin 1), and CD47 (CD47 Molecule). These AS genes are also validated in cell samples over-expressed by CELF6 (Fig. 4G). CELF6-affected TP53 and CD44 at the AS levels are associated with apoptosis, migration signaling pathways, which are associated with oncogenesis, cancer metastasis, prognosis, and total survival in many cancers (Han et al., 2003; Li et al., 2016; Roudi et al., 2014). CD44 is a cell-surface glycoprotein involved in cell-cell interactions, cell adhesion and migration. High expression of CD44 is not only associated with the occurrence and migration of NSCLC (Li et al., 2016; Liu et al., 2015), but also with drug resistance and poor prognosis (Liu et al., 2013; Shinohara et al., 2016). In NSCLC, CD44 has multiple variable shear subtypes, and the expression of cell-adhesion associated with CD44v6 is highly relevant to the lymph node metastasis of NSCLC (Su et al., 2014).

It is well known that P53 signaling pathways are an important biological process that affects cancer cell apoptosis. Recent studies have reported that vinorelbine combined with afatinib can induce apoptosis in non-small cell lung cancer by activating p53-related signaling pathways (Liu et al., 2021). TP53 is known to be a transcription factor. TP53 is related to the progression of NSCLC. The expression of TP53 is related to the aggressiveness of cancer. The mutation of TP53 affects the poor prognosis of non-small cell lung cancer (Canale et al., 2020; Qin et al., 2020). Silencing different splicing factors seems to activate p53 in different ways, although p53 levels are increased in almost all cases (Siebring-van Olst et al., 2017). Over-expression of another RBP RBM10 induces p53-dependent apoptosis in various cancer cells (Jung et al., 2020), indicating that CELF6 is likely to contribute to tumorigenesis *via* regulating the AS of TP53 or CD44 in non-small cell lung cancer cells.

## Conclusions

In conclusion, we successfully applied RNA-seq technology to prove that CELF6 regulates alternative splicing, and that the functions of splicing factors are associated with tumor immunity. We found that in A549 cells, CELF6 may regulate tumorigenesis and cancer progression by repressing the expression of immune response genes. Our results also emphasize that the well-known splicing factor CELF6 may activate the P53 signaling pathway by regulating the alternative splicing of its key genes. Further research on CELF6-regulated alternative splicing would be helpful to accurately understand the signaling network that guides the occurrence of non-small cell lung cancer, as well as the potential of CELF6-targeting therapies.

## Supplemental Information

10.7717/peerj.13800/supp-1 Supplemental Information 1 Uncropped gels for WB.

10.7717/peerj.13800/supp-2 Supplemental Information 2 (A) The top 10 representative GO biological processes of up-regulated genes. (B) Bar plot exhibited the most enriched KEGG pathways results of the up-regulated and down-regulated DEGs.

10.7717/peerj.13800/supp-3 Supplemental Information 3 CELF6 regulated alternative splicing of SEC31A (A), FN1(B) and CD47(C). **Left:** IGV-sashimi plot showing the regulated alternative splicing events and binding sites across mRNA. Reads distribution of RASE is plotted in the up panel and the transcripts of each gene are shown below. **Right:** The schematic diagrams depict the structures of ASEs. RNA-seq validation of ASEs are shown at the bottom of the right panel. Error bars represent mean ± SEM. ***P-value < 0.001, ** *P*-value < 0.01, * *P*-value < 0.05.

10.7717/peerj.13800/supp-4 Supplemental Information 4 Primer sequences used for RT-qPCR.

10.7717/peerj.13800/supp-5 Supplemental Information 5 Raw and processed data of RT-qPCR results.

10.7717/peerj.13800/supp-6 Supplemental Information 6 The details of the DEGs.

10.7717/peerj.13800/supp-7 Supplemental Information 7 GO analysis of Up-regulated genes.

10.7717/peerj.13800/supp-8 Supplemental Information 8 KEGG analysis of Up-regulated genes.

10.7717/peerj.13800/supp-9 Supplemental Information 9 GO analysis of Down-regulated genes.

10.7717/peerj.13800/supp-10 Supplemental Information 10 KEGG analysis of Down-regulated genes.

10.7717/peerj.13800/supp-11 Supplemental Information 11 Annotated splice junctions and novel splice junctions were detected using TopHat2 software.

10.7717/peerj.13800/supp-12 Supplemental Information 12 Classification of all regulated alternative splicing events.

## Additional Information and Declarations

### Data Availability

The following information was supplied regarding data availability: The data is available at NCBI GEO: GSE185865.
