# Supplementary figures and images for: RNA-binding protein CELF6 modulates transcription and splicing levels of genes associated with tumorigenesis in lung cancer A549 cells
Source: PeerJ. 2022 Jul 26;10:e13800. doi: 10.7717/peerj.13800 (PMC9336609; doi:10.7717/peerj.13800)

A

Ctrl1 Ctrl2 OE1 OE2

70 kDa

50 kDa

CELF6-FLAG

40 kDa

35 kDa

GAPDH

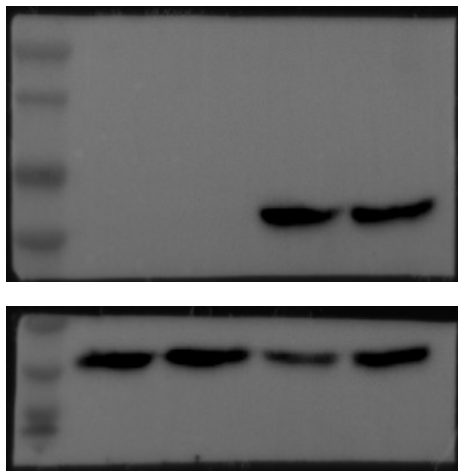

Supplement: Supplemental Information 1 [file peerj-10-13800-s001.pdf]

A

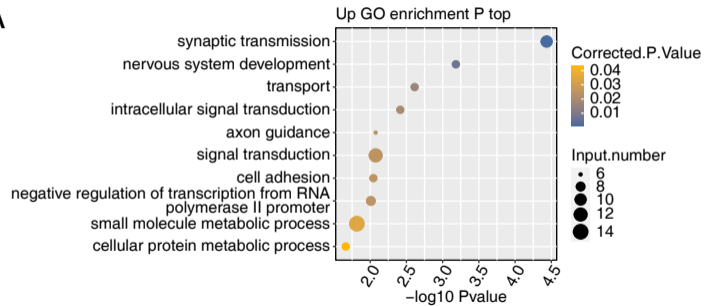

B

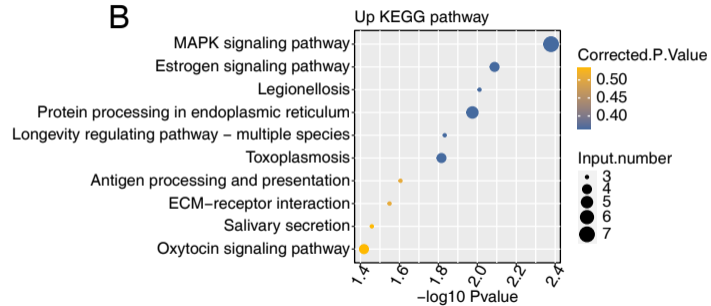

Supplement: Supplemental Information 2 [file peerj-10-13800-s002.pdf]

A

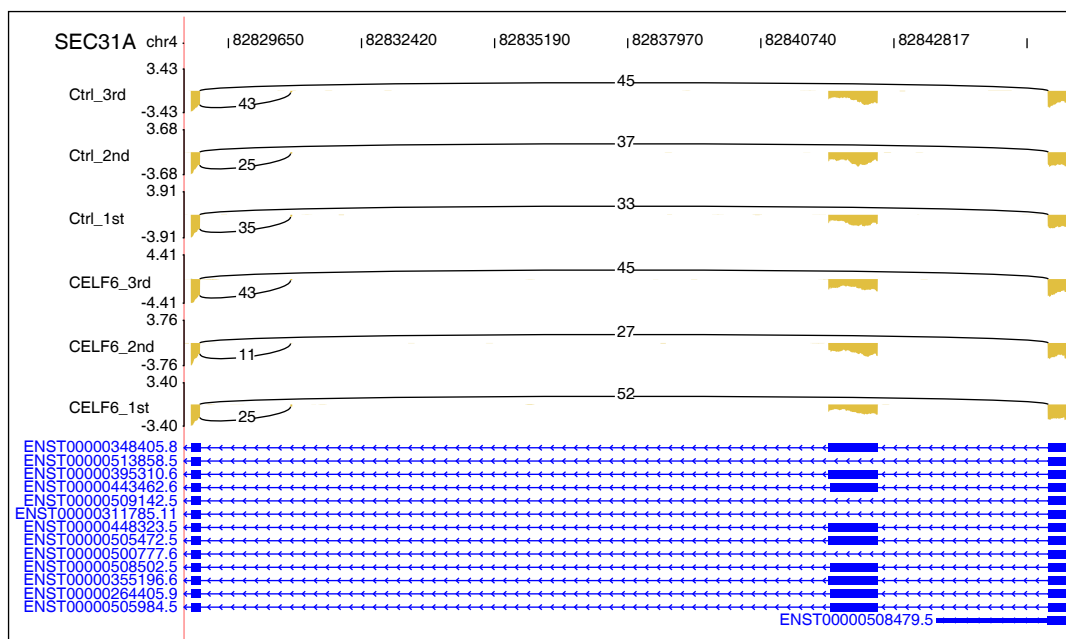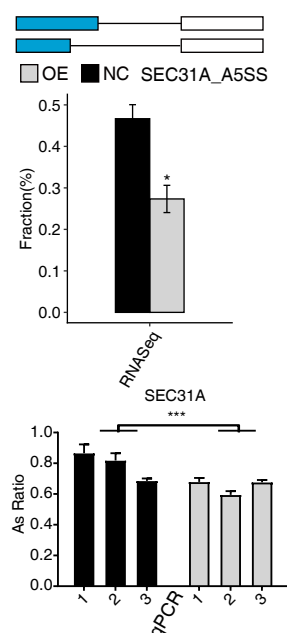

B

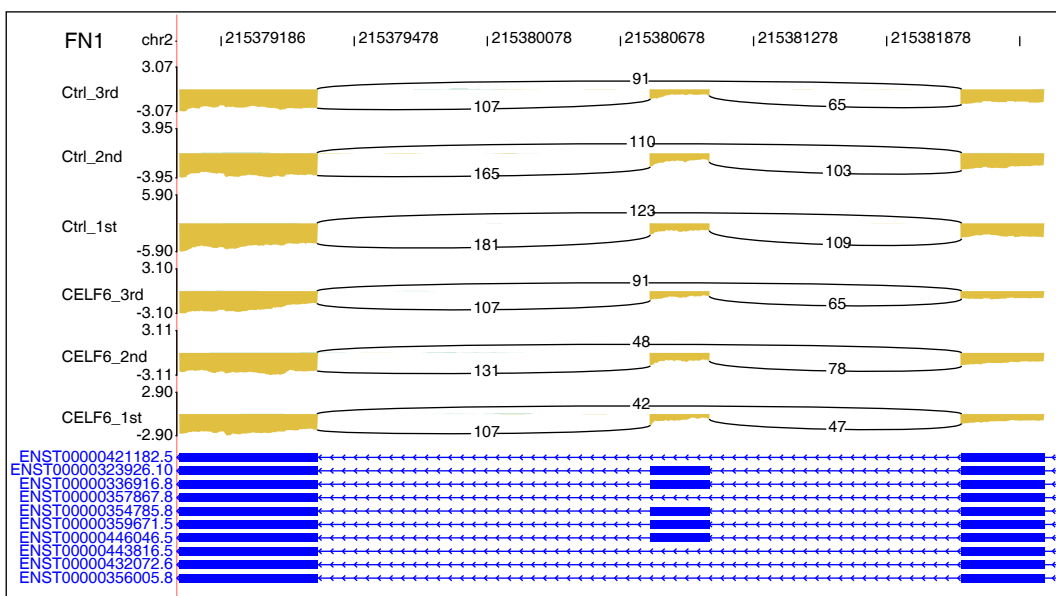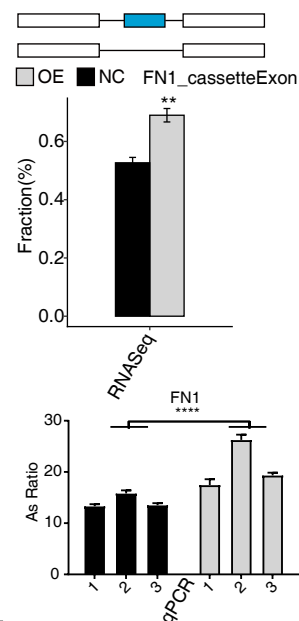

C

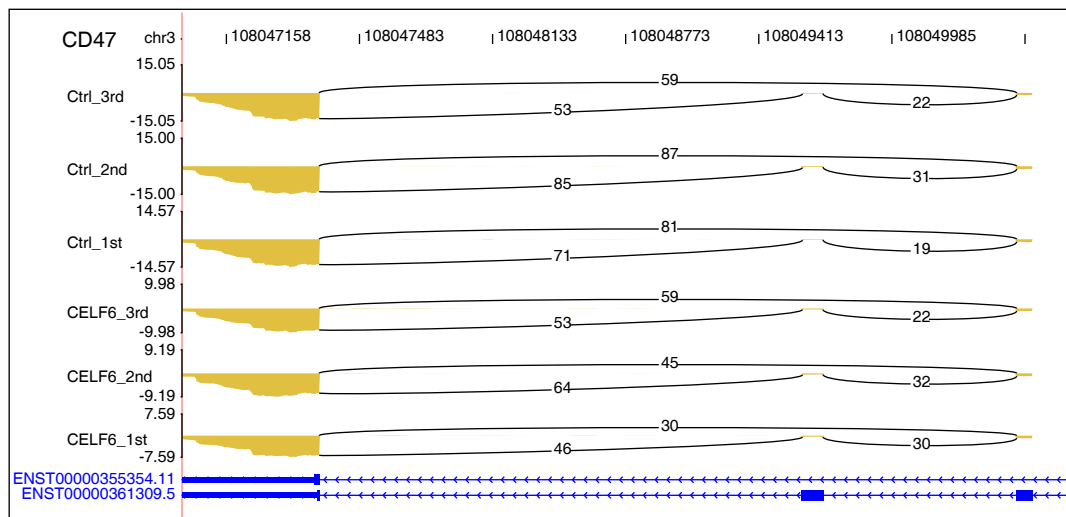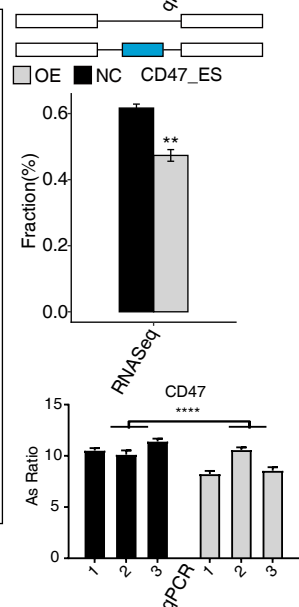

Supplement: Supplemental Information 3 — Left: IGV-sashimi plot showing the regulated alternative splicing events and binding sites across mRNA. Reads distribution of RASE is plotted in the up panel and the transcripts of each gene are shown below. Right: The schematic diagrams depict the structures of ASEs. RNA-seq validation of ASEs are shown at the bottom of the right panel. Error bars represent mean ± SEM. ***P-value < 0.001, ** P-value < 0.01, * P-value < 0.05. [file peerj-10-13800-s003.pdf]
